# First line immunotherapy extends brain metastasis free survival, improves overall survival, and reduces the incidence of brain metastasis in patients with advanced melanoma

**DOI:** 10.1002/cnr2.1419

**Published:** 2021-06-17

**Authors:** Xuechen Wang, Benjamin Haaland, Siwen Hu‐Lieskovan, Howard Colman, Sheri L. Holmen

**Affiliations:** ^1^ Huntsman Cancer Institute University of Utah Health Sciences Center Salt Lake City Utah USA; ^2^ Department of Population Health Sciences University of Utah Salt Lake City Utah USA; ^3^ Department of Internal Medicine University of Utah Health Sciences Center Salt Lake City Utah USA; ^4^ Department of Neurosurgery University of Utah Health Sciences Center Salt Lake City Utah USA; ^5^ Department of Surgery University of Utah Health Sciences Center Salt Lake City Utah USA; ^6^ Department of Oncological Sciences University of Utah Health Sciences Center Salt Lake City Utah USA

**Keywords:** first line therapy, immunology, melanoma, metastasis, target therapy

## Abstract

**Background:**

Recent advances in targeted therapy and immunotherapy have improved the prognosis of melanoma patients but brain metastasis remains a major challenge. Currently, it is unclear how existing therapies can be best used to prevent or treat brain metastasis in melanoma patients.

**Aims:**

We aimed to assess brain metastasis free survival (BMFS), overall survival (OS), incidence of brain metastases, and sequencing strategies of immunotherapy and targeted therapy in patients with BRAF‐mutated advanced melanoma.

**Methods and results:**

We retrospectively analyzed 683 patients with BRAF‐mutated advanced melanoma treated with first line (1L) immunotherapy (*N* = 266) or targeted therapy (*N* = 417). The primary outcome was BMFS. Secondary outcomes included OS of all patients and incidence of brain metastases in patients without documented brain metastases prior to 1L therapy. The median BMFS was 13.7 months [95% confidence interval (CI): 12.4–16.0] among all patients. The median BMFS for patients receiving 1L immunotherapy was 41.9 months [95% CI: 22.8–not reached (NR)] and targeted therapy was 11.0 months (95% CI: 8.8–12.5). Median OS results were qualitatively similar to BMFS results. The cumulative incidence of brain metastases for patients receiving 1L targeted therapy was higher than for patients receiving 1L immunotherapy (*P* < .001). Patients receiving 1L anti‐CTLA4 plus anti‐PD1 combination immunotherapy only or followed by second line (2L) targeted therapy had better BMFS (HR 0.40, 95% CI: 0.24–0.67, *P* = .001), improved OS (HR 0.49, 95% CI: 0.30–0.81, *P* = .005), and reduced incidence of brain metastases (HR 0.47, 95% CI: 0.24–0.67, *P* = .047) than patients receiving 1L combination BRAF and MEK targeted therapy followed by 2L immunotherapy.

**Conclusion:**

Patients with advanced BRAF mutant melanoma treated with 1L immunotherapy have significantly longer BMFS and OS, and reduced incidence of brain metastases, compared with those treated with 1L targeted therapy. Further studies evaluating the ability of immunotherapy and targeted therapy to improve OS and prevent brain metastases are warranted

## 1 INTRODUCTION

Melanoma is noted for its continued increase in incidence and propensity to metastasize to distant organs. More than 106 000 new cases are expected in the United States (U.S.) in 2021,^1^ which is more than double the number of new melanoma cases in 2001.^2^ Melanoma mortality rates also continued to increase until 2017.^3^ This recent decline in mortality is likely due to the significant advances in treatment of advanced melanoma that has occurred over the past decade. These include Food and Drug Administration (FDA) approved therapies consisting of ipilimumab, an anti‐cytotoxic T‐lymphocyte‐associated antigen 4 (CTLA‐4) humanized monoclonal antibody (mAb),^4^ the pharmacological v‐raf murine sarcoma viral oncogene homolog B1 BRAF^V600E^ inhibitor vemurafenib,^5^ the MEK1/2 inhibitor trametinib,^6^ the combination of the BRAF^V600E^ inhibitor dabrafenib and trametinib^7^ or the BRAF^V600E^ inhibitor encorafenib and MEK1/2 inhibitor binimetinib,^8^ mAbs targeting programmed cell death protein 1 (PD1), pembrolizumab and nivolumab,^8^ the combination of ipilimumab plus nivolumab, the combination of vemurafenib and the MEK1/2 inhibitor cobimetinib,^9^ and talimogene laherparepvec (T‐VEC), the first oncolytic virus therapy.^10^ More recently, the FDA approved ipilimumab,^11^ nivolumab,^12^ pembrolizumab,^13^ and the dabrafenib plus trametinib combination (*BRAF*‐mutated patients only) for adjuvant therapy of high‐risk melanoma, based on significantly longer recurrence‐free survival associated with these therapeutic interventions.^14^

Despite these advances, brain metastases remain a major complication of metastatic melanoma and are responsible for up to half of all melanoma deaths,^15^, ^16^, ^17^, ^18^ with median overall survival (OS) of less than 2 years.^19^ Among all cancers that frequently metastasize to the brain, including breast, lung, colon, and kidney, melanomas have the highest frequency for colonizing this organ.^20^, ^21^, ^22^, ^23^ In the largest brain specific targeted therapy trial to date in patients with active brain metastases, COMBI‐MB (dabrafenib plus trametinib), intracranial response rates were 58% but most responses were short‐lived (median duration 6.5 months) compared with the same drugs in patients without brain metastases (12.9 months) and most treatment failures occurred in the brain.^24^ A phase II trial of ipilimumab, which included patients with symptomatic and asymptomatic brain metastases, found that 10% and 24% achieved partial response or stable disease, and median OS of 3.7 and 7 months, respectively.^25^ In a phase II clinical trial of pembrolizumab for patients with asymptomatic melanoma brain metastases, the intracranial overall response rate (ORR) was 26% and progression‐free survival (PFS) and 2‐year OS were 2 and 17 months, respectively.^26^ Recently, CheckMate‐204 assessed ipilimumab in combination with nivolumab followed by nivolumab as single agent for previously untreated patients with at least one active brain metastasis and no steroid use.^27^ The systemic ORR was 57%. It is important to note that at the time of publication, the median PFS and duration of response had not yet been reached for responding patients. A separate multi‐center, randomized trial evaluated the combination of nivolumab plus ipilimumab versus nivolumab alone in a similar patient population. Patients with asymptomatic melanoma brain metastases demonstrated an intracranial objective response rate of 46% for the combination and 20% for nivolumab alone.^28^ While these studies suggest that immunotherapy can provide durable responses for some melanoma patients with brain metastases, they also reveal the critical need for new therapeutic strategies for those patients who remain refractory. Furthermore, it remains unclear, which therapy is best in the first‐line (1L) setting and if the order of these therapies affects the development of brain metastases and/or OS. Although treatment sequencing is being addressed in the Doublet Randomized Evaluation in Advanced Melanoma Sequencing (DREAMseq; EA6134) Phase III prospective trial, study completion is estimated to be late 2022. Presently, there is limited data available to guide clinicians and patients in choosing between these options.

In this study, we utilized the Flatiron Health database to retrospectively assess brain metastasis free survival (BMFS) from time of initiation of 1L therapy (immunotherapy, or targeted therapies) to metastasis or death in patients with advanced BRAF mutant melanoma. We also assessed OS, incidence of brain metastases, and compared sequencing strategies of immunotherapy and targeted therapies, comparing 1L immunotherapy with 1L targeted therapy in patients with or without brain metastases.

## 2 METHODS

### 2.1 Data

Patients with advanced melanoma were identified via the Flatiron Health database, a nationwide longitudinal, demographically, and geographically diverse database derived from de‐identified electronic health record (EHR) data from over 280 community‐based cancer treatment clinics and academic centers representing about 2.4 million U.S. cancer patients. Patients with pathologic stage III or IV melanoma at initial diagnosis on or after January 1, 2011, or with earlier stage disease who developed a first locoregional or distant recurrence on or after January 1, 2011, were considered advanced. The analysis cohort consisted of patients with EHR documentation of a BRAF mutation positive lesion who received 1L treatment with single agent anti‐CTLA4 immunotherapy, single agent anti‐PD1 immunotherapy, combination anti‐CTLA4/anti‐PD1 immunotherapy, targeted BRAF inhibition, or combined targeted BRAF/MEK inhibition. Patients were included if they initiated 1L therapy on one of the treatments of interest from January 1, 2011, to February 28, 2019, to ensure at least 6 months of potential follow‐up. Patients were also required to have at least one documented clinic visit within 90 days after advanced diagnosis date, in order to ensure that they were primarily engaged with the relevant practice. Patients who developed a brain metastasis before the initiation of 1L treatment were excluded. Analyses were based on de‐identified data. Institutional review board approval of the study protocol was obtained prior to study conduct.

### 2.2 Outcomes

The primary outcome was BMFS from time of 1L initiation to brain metastasis or death. Secondary outcomes were OS and incidence of brain metastases from time of 1L initiation. Patients were right censored at the end of last known follow‐up or hospice referral if the mortality or metastatic event of interest was unknown or had not yet occurred.

### 2.3 Statistical analyses

Baseline patient characteristics were determined based on the most recent EHR documentation from 1 month prior to advanced or metastatic diagnosis until initiation of 1L therapy. If the characteristic of interest was not documented in the EHR within the above time window, then the corresponding data value was encoded in a separate “Missing” category. Baseline characteristics were compared between patients receiving different 1L treatments using Wilcoxon rank sum test or Chi‐squared tests, as appropriate. BMFS and OS were summarized via Kaplan–Meier, and incidence of brain metastases was summarized via cumulative incidence subject to competing risk due to death.^29^ The main comparisons among treatments were based on matching weighed^30^ Cox proportional hazards models using for time‐to‐event endpoints,^31^ and matching weighed^30^ Fine and Gray models for incidence of brain metastases.^32^ Propensity scores were constructed via random forest^33^ out‐of‐bag predictions based on gender, race, ethnicity, age at advanced or metastatic diagnosis, disease stage at initiation of 1L therapy, 1L start year, and baseline Eastern Cooperative Oncology Group (ECOG), bilirubin, Alanine Aminotransferase (ALT), Aspartate Transaminase (AST), Lactate Dehydrogenase (LDH), and Estimated Glomerular Filtration Rate (eGFR). Analyses were performed in R 3.6.0 and SAS version 9.4.^34^

The primary comparison was between immunotherapy (single agent or combination) and targeted therapies (single agent or combination). Secondary comparisons included comparisons within subgroups, pairwise comparisons of specific regimens, and comparison of sequencing strategies. Comparisons of specific treatments were conducted within time windows reflecting widespread availability and adoption of both agents. Treatment sequences were compared targeting an intention‐to‐treat analysis, where patients may have the event of interest or drop‐out prior to second‐line (2L) initiation, and these patients were included in the corresponding sequence arm.

## 3 RESULTS

### 3.1 Patients

A total of 683 patients with advanced (stage III) or metastatic (stage IV) melanoma and EHR documented evidence of BRAF mutated lesions treated with 1L immunotherapy anti‐CTLA4/anti‐PD1/combo (*n* = 266), or targeted BRAF/combo (*n* = 417), who did not have brain metastases at 1L initiation, were identified and included in our primary study. The median patient age was 62 (interquartile range [IQR]: 53–72) years. The median follow‐up time was 12.3, 15.6, and 10.9 months for patients receiving any treatment, immunotherapy, or targeted therapy, respectively (Table 1). Over the time range 2011–2019, the proportion of patients receiving immunotherapy increased, while the proportion receiving targeted therapy decreased. The median baseline LDH level was 219.5 (IQR: 168.0–427.5) units/L, while patients who received targeted therapy had significantly higher median LDH level than those who received immunotherapy (254 vs. 193.5, *P* < .001). Normal LDH levels range from 140 U/L to 280 U/L. Among patients with stage IV disease, 62.8% received targeted therapy and only 37.2% received immunotherapy. However, of patients with stage III disease, 50.5% and 49.5% received targeted therapy or immunotherapy, respectively. Among patients with a recorded baseline ECOG performance status 0–1 (86.8%), 53.4% received 1L targeted therapy and 46.6% received 1L immunotherapy, whereas among patients with ECOG ≥2, 66.0% received 1L targeted therapy and 34.0% received 1L immunotherapy. All patients with documented positive PDL1 expression (12 out of 45 tested patients) received 1L immunotherapy.

**TABLE 1:** Patient characteristics by the first‐line treatments for BRAF mutated patients

Characteristic		BRAF mutated			
		Overall (*N* = 683)	Immunotherapy (*N* = 266)	Targeted therapy (*N* = 417)	*P*‐value
Practice type: Academic		87 (100%)	47 (54.0%)	40 (46.0%)	*P* = .003
First line start year	2011‐2013	119 (100%)	9 (7.6%)	110 (92.4%)	*P* < .001
	2014‐2016	326 (100%)	131 (40.2%)	195 (59.8%)	
	2017‐2019	238 (100%)	126 (52.9%)	112 (47.1%)	
ECOG	0–1	309 (100%)	144 (46.6%)	165 (53.4%)	*P* = .146
	≥2	47 (100%)	16 (34.0%)	31 (66.0%)	
PDL1	Positive	12 (100%)	12 (100.0%)	0 (0.0%)	*P* < .001
	Negative	33 (100%)	18 (54.5%)	15 (45.5%)	
	Not Tested	628 (100%)	229 (36.5%)	399 (63.5%)	
	Unknown	10 (100%)	7 (70.0%)	3 (30.0%)	
Stage	Stage IV	588 (100%)	219 (37.2%)	369 (62.8%)	*P* = .031
	Stage III	95 (100%)	47 (49.5%)	48 (50.5%)	
LDH		Median 219.5 (IQR 168.0–427.5)	Median 193.5 (IQR 162.8‐302.2)	Median 254.0 (IQR 184.2‐482.8)	*P* < .001
Age at advanced diagnosis		Median 62.0 (IQR 53.0‐72.0)	Median 63.0 (IQR 54.0‐71.8)	Median 62.0 (IQR 52.0‐72.0)	*P* = .655
Follow‐up time		Median 12.3 (IQR 5.5‐26.4)	Median 15.6 (IQR 6.5‐30.5)	Median 10.9 (IQR 5.3‐23.5)	*P* = .008

### 3.2 Treatment outcomes

**FIGURE 1:**
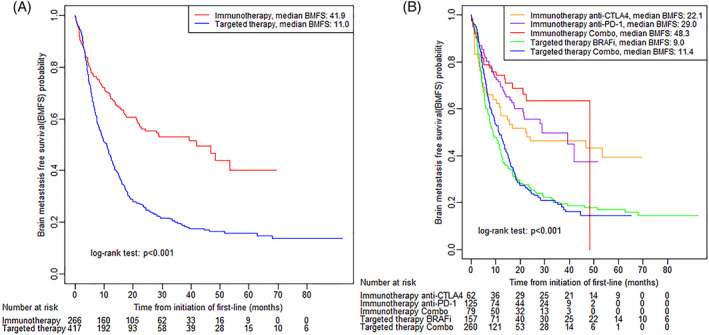
Kaplan–Meier curves of brain metastasis free survival for BRAF mutated patients who did not have brain metastases at 1L initiation by first‐line treatments (A), and by break‐down of first‐line treatments (B)

More than half of the patients received 1L therapy only, and 18.6% received three or more lines of therapy. Among our study cohort, there were 44.6% of patients with an EHR documented new metastasis after 1L therapy initiation. Among patients receiving 1L immunotherapy or targeted therapy, 16.5% and 30.9% developed a brain metastasis, respectively. The median BMFS was 13.7 months [95% confidence interval (CI): 12.4–16.0] among all patients, 41.9 months [95% CI: 22.8–not reached (NR)] for patients receiving 1L immunotherapy, and 11.0 months [95% CI: 8.8–12.5] for targeted therapy (Table 2). Patients receiving 1L immunotherapy had significantly longer BMFS compared with patients receiving targeted therapy (unadjusted log‐rank *P* < .001, Figure 1(A)). BMFS was numerically longer for patients receiving combination immunotherapy relative to patients receiving single agent immunotherapy, but the differences were not statistically significant (*P* = .093 and *P* = .354, respectively, for combination immunotherapy vs. single agent CTLA4 and single agent PD1 inhibition, Figure 1(B)). There was also no evidence indicating that BMFS was significantly different between patients receiving combination targeted therapy and patients receiving single agent BRAF inhibition (*P* = .567, Figure 1(B)). Results were qualitatively similar for OS (Figure S1). The cumulative incidence of brain metastases for patients receiving 1L targeted therapy was higher than for patients receiving 1L immunotherapy (*P* < .001, Figure S2(A)). Visually, the corresponding two lines separated after 5 months from initiation of 1L therapy. The cumulative incidence of brain metastasis was higher for patients receiving 1L anti‐CTLA4 immunotherapy, compared with patients receiving either 1L anti‐PD‐1 immunotherapy or combination immunotherapy (*P* = .043, Figure S2(B)).

**TABLE 2:** Patient outcomes by the first‐line treatments for BRAF mutated patients (NR: not reached)

		Overall (*N* = 683)	BRAF mutated		
			Immunotherapy (*N* = 266)	Targeted therapy (*N* = 417)	*P*‐value
Number of lines	1	379 (55.5%)	165 (62.0%)	214 (51.3%)	*P* = .016
	2	177 (25.9%)	59 (22.2%)	118 (28.3%)	
	3	80 (11.7%)	22 (8.3%)	58 (13.9%)	
	≥4	47 (6.9%)	20 (7.5%)	27 (6.5%)	
Metastasis after first line	Brain	173 (25.3%)	44 (16.5%)	129 (30.9%)	*P* < .001
	None	378 (55.3%)	173 (65.0%)	205 (49.2%)	
	Other	132 (19.3%)	49 (18.4%)	83 (19.9%)	
Brain metastasis free survival (median [95%CI])		13.7 (12.4–16.0)	41.9 (22.8 – NR)	11.0 (8.8–12.5)	*P* < .001
Overall survival (median [95% CI])		19.5 (16.8‐23.5)	48.3 (28.8–NR)	13.8 (11.5‐17.0)	*P* < .001

### 3.3 Comparative effectiveness

**FIGURE 2:**
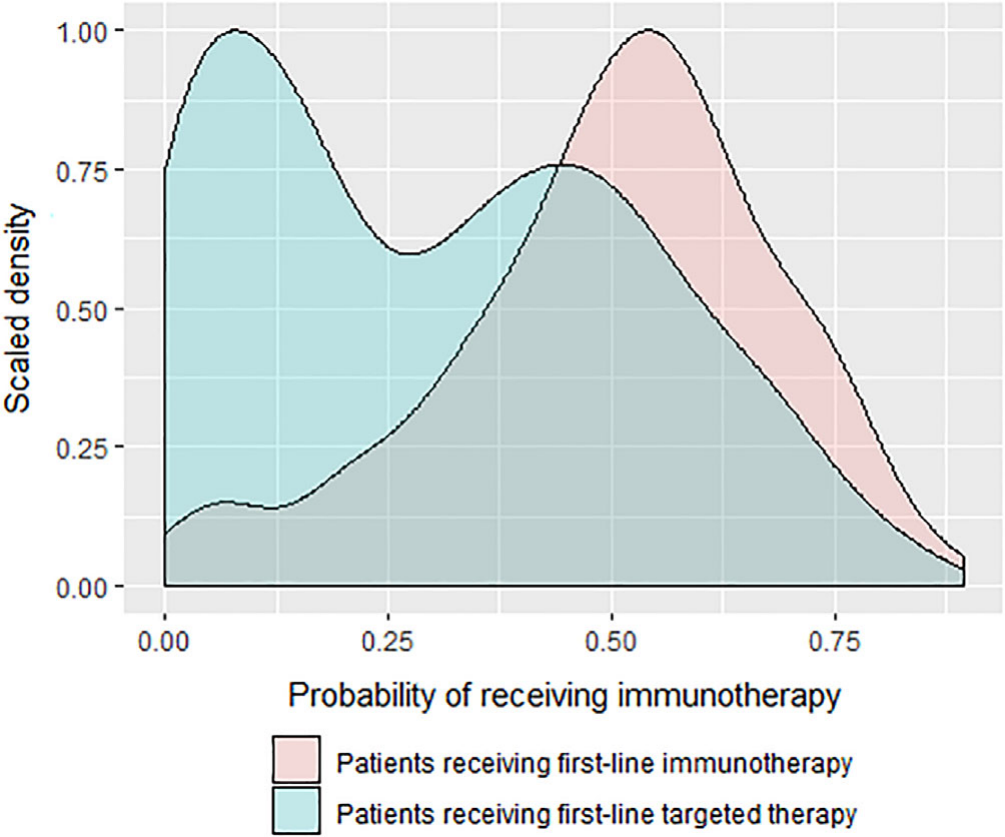
Distributions of predicted probability of receiving first‐line immunotherapy by first‐line treatment groups, which are used to construct the treatment propensity scores (probability of treatment actually received). The *x*‐axis shows the range of predicted probabilities, and the *y*‐axis shows the scaled density. Patients receiving different first‐line treatments are represented by different colors. The overlapping region indicates patients receiving different first‐line treatments were comparable because they had similar predicted probability of receiving immunotherapy

For the main comparisons, propensity distributions were strongly overlapping, indicating the feasibility of comparisons of immunotherapy and targeted therapy (Figure 2). Matching weights were obtained based on the propensity scores. After matching weighting, treatment groups were generally well‐balanced with respect to potential confounders (which were used in the propensity score model). However, 1L start year [matching weighted standardized mean difference (SMD) 0.245, *P* = .025], ALT (matching weighted SMD 0.153, *P* = .059), AST (matching weighted SMD 0.150, *P* = .060), and disease stage at initiation of 1L therapy (matching weighted SMD 0.140, *P* = .123) remained partially imbalanced after matching weighting, and these variables were included as covariates in the primary matching weighted comparative effectiveness models. In particular, even after matching weighting, patients tended to receive 1L targeted therapy during or before 2015, and received 1L immunotherapy after 2015; also the average ALT and AST were higher for patients receiving targeted therapy. The matching weighted Cox proportional hazards (PH) model indicated that immunotherapy leads to better BMFS outcomes [hazard ratio (HR) 0.51, 95% CI: 0.40–0.66, *P* < .001] and better OS outcomes (HR 0.56, 95% CI: 0.43–0.73, *P* < .001) than targeted therapy (Table 3 and Figure S3). The matching weighted Fine and Gray model indicated that immunotherapy also leads to reduced incident brain metastasis outcomes relative to targeted therapy (HR 0.51, 95% CI: 0.34–0.77, *P* = .002; Figure S4).

**TABLE 3:** Matching weighted comparative effectiveness for first‐line immunotherapy versus first‐line targeted therapy across endpoints

Ref: Targeted therapy	Hazard ratio (95% CI, *P*‐value)
Immunotherapy	
Brain metastasis free survival	0.51 (0.40–0.66, *P* < .001)
Overall survival	0.56 (0.43–0.73, *P* < .001)
Incident brain metastases	0.51 (0.34–0.77, *P* = .002)

### 3.4 Subgroup analyses

**FIGURE 3:**
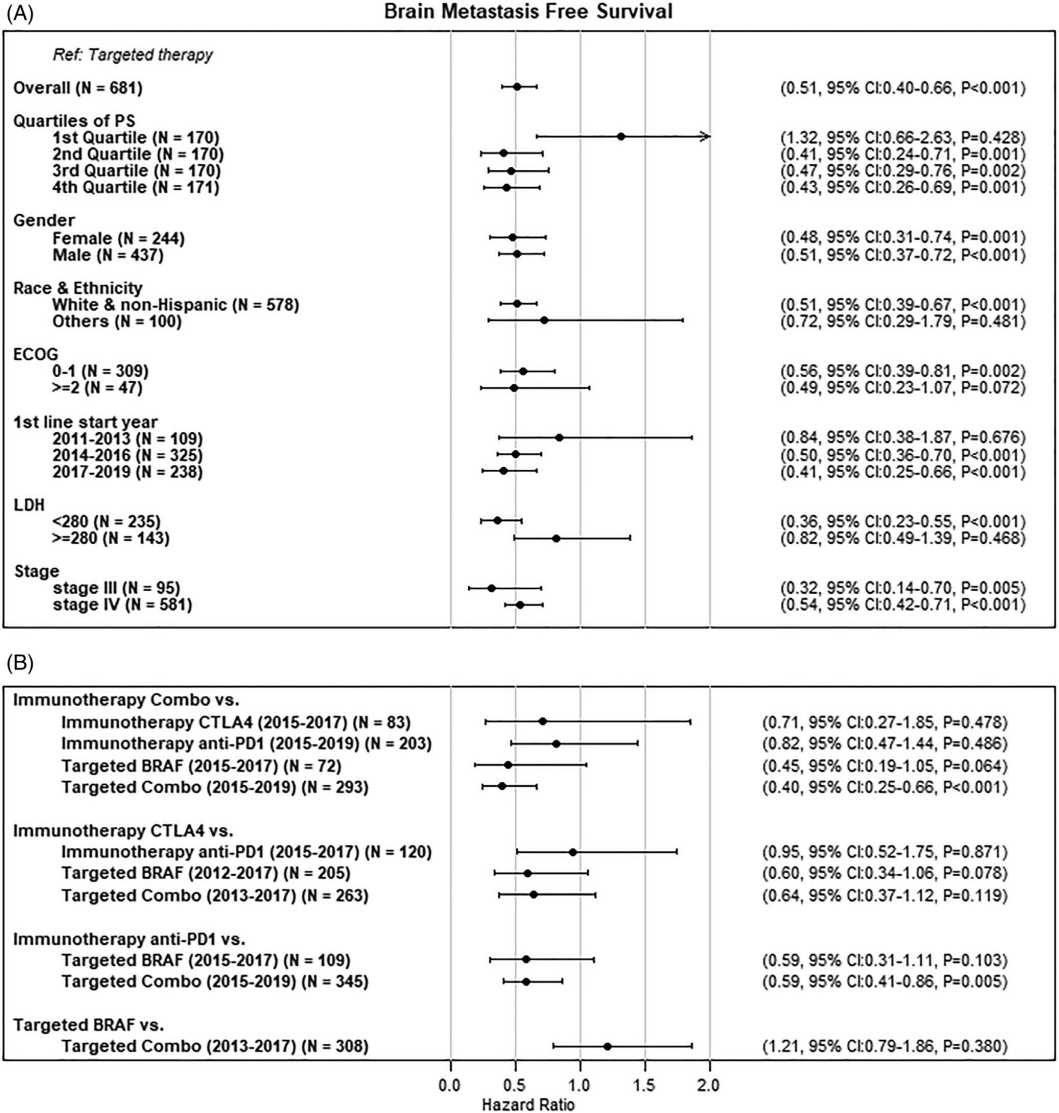
Forest plots of hazard ratios of brain metastasis‐free survival from matching weighted Cox PH models for subgroups of patients (A), and for pairwise of treatment regimens (B). PS is the probability of receiving immunotherapy. The date ranges of the figure correspond to when both treatments of the comparisons are available

**FIGURE 4:**
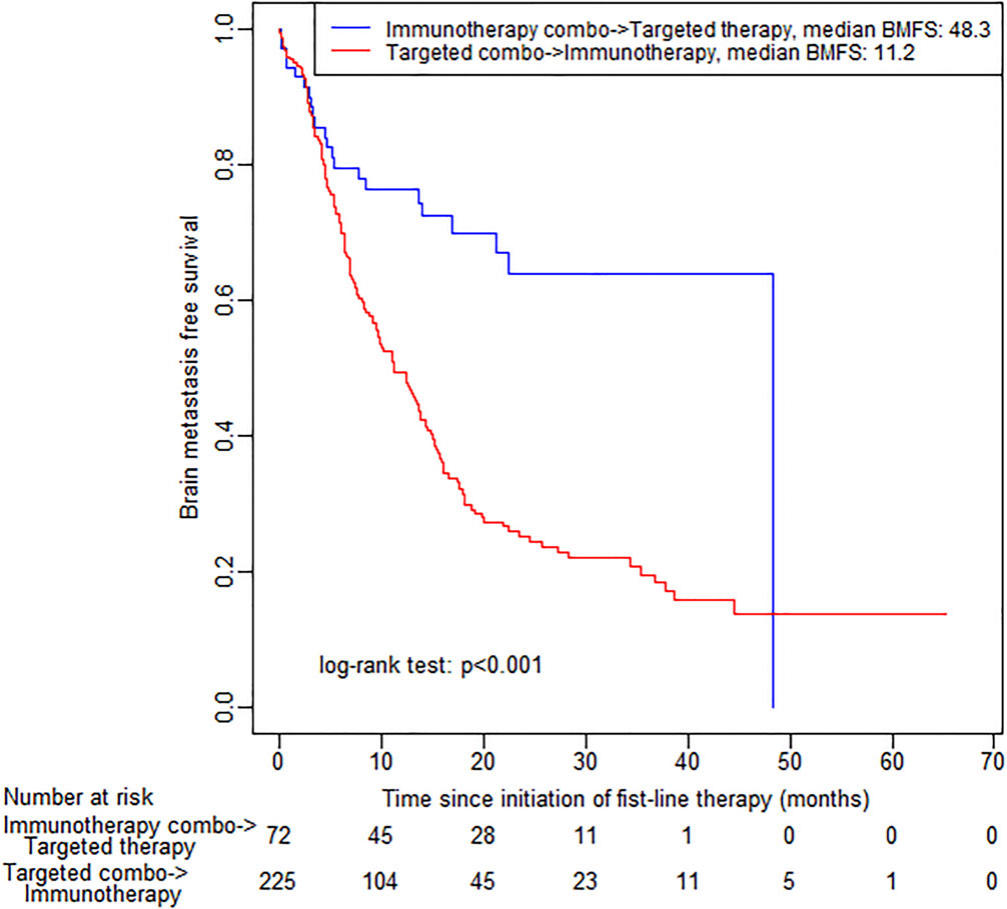
Kaplan–Meier curves by treatment sequences for brain metastasis free survival

Matching weighted HRs for subgroups of interest are shown in Figure 3A and Figures 3(A) and 4(A). Comparisons of immunotherapy to targeted therapy were relatively consistent across subgroups. Notably, subgroup comparisons suggested that the beneficial effect of immunotherapy on BMFS may be greater for patients with a greater propensity for receiving immunotherapy, indicating that providers are using patient characteristics to prescribe immunotherapy among patients most likely to receive the greatest benefit. Subgroup comparisons also suggested that the benefits of immunotherapy over targeted therapy may be greater in more recent years, for patients with a better prognosis (LDH < 280 units/L), and for patients with stage III compared with stage IV. Subgroup comparisons suggested that the efficacy of immunotherapy relative to targeted therapy was similar for males and females (Figure 3(A)). Subgroup comparisons for OS and incident brain metastases were qualitatively similar. We did not observe significant beneficial impact of immunotherapy relative to targeted therapy for patients with ECOG > 1, which may be due to the limited number of patients within that group (Figures S3(A) and S4(A)). In addition, the variance of HRs for incident brain metastases was relatively larger compared with other outcomes.

### 3.5 Pairwise comparisons

Matching weighted HRs for specific treatment comparisons are shown in Figure 3B and Figures S3(B) and S4(B). Pairwise comparisons of the five 1L treatments' effects on BMFS suggested that combination immunotherapy may be the most effective treatment, followed by single agent immunotherapy (anti‐PD‐1 then anti‐CTLA4) and then targeted therapy. The effects of combination targeted therapy and single agent BRAF inhibition was similar (Figure 3B). Pairwise comparison results were similar for OS; however, the results for incident brain metastases had higher variability (Figures S3(B) and S4(B)).

### 3.6 Sequencing

**FIGURE 5:**
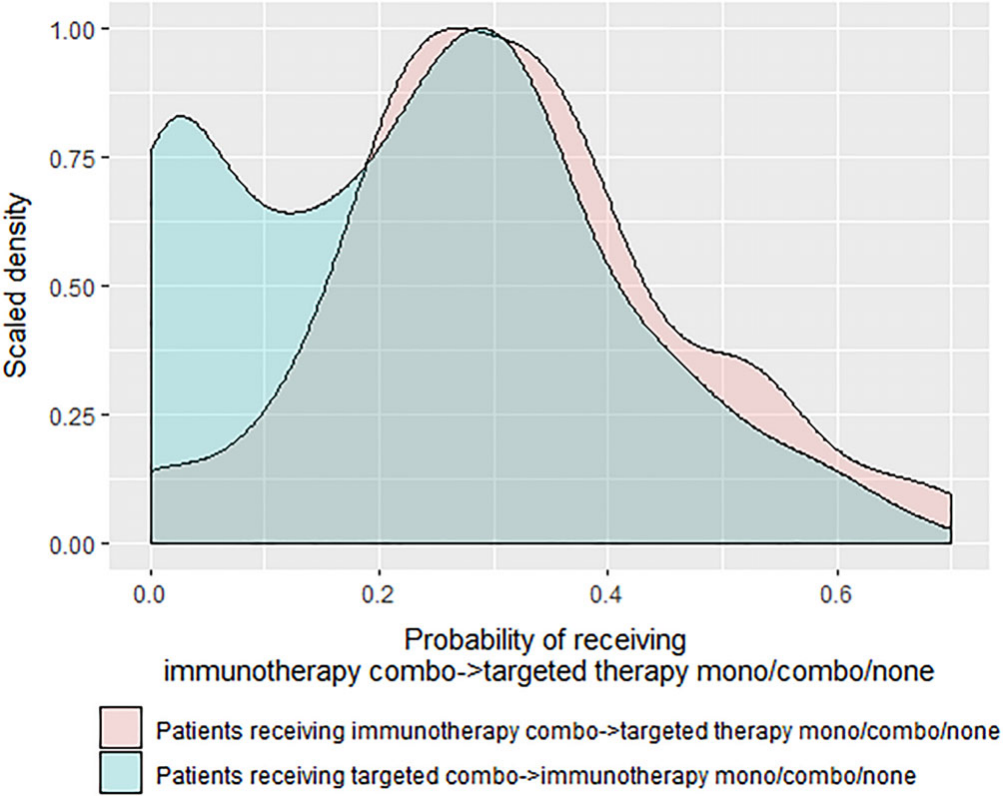
Distributions of predicted probability of receiving first‐line combination immunotherapy followed by second‐line targeted therapy or no second‐line therapy by treatment sequence, which are used to construct the treatment sequence propensity scores (probability of treatment sequence actually received). The *x*‐axis shows the range of predicted probabilities, and the *y*‐axis shows the scaled density. Patients receiving different treatment sequences are represented by different colors. The overlapping region indicates patients receiving different treatment sequences were comparable because they had similar predicted probability of receiving first‐line combination immunotherapy followed by second‐line targeted therapy or no second‐line therapy

Among the 683 BRAF mutated patients, 72 patients (24.2%) received 1L combination immunotherapy followed by 2L targeted therapy (including combination targeted therapy and single agent BRAF inhibition) or no 2L therapy; 225 patients (75.8%) received 1L combination targeted therapy followed by 2L immunotherapy (including combination immunotherapy and single agent CTLA4 or PD1 immunotherapy) or no 2L therapy (Table 4). The median BMFS was 48.3 months for patients with 1L combination immunotherapy followed by 2L targeted therapy or no 2L therapy, and 11.2 months for patients with 1L combination targeted therapy followed by 2L immunotherapy or no 2L therapy (Figure 4). Results were similar for OS and incident brain metastases (Figures S5 and S6). The 75th percentile of BMFS was 13.6 (95% CI: 4.6–NR) months, and 75th percentile of OS was 16.9 (95% CI: 6.5–NR) months for patients with 1L combination immunotherapy followed by 2L targeted therapy or no 2L therapy. Propensity distributions for these 297 patients are shown in Figure 5. The strongly overlapping propensity distributions suggest the feasibility of comparing the two treatment sequencing strategies. After matching weighting, variables, which were not well‐balanced were included as covariates in the matching weighted comparative effectiveness models. The analyses results suggest that the sequencing strategy of 1L combination immunotherapy followed by 2L targeted therapy/none leads to better BMFS outcomes than 1L combination targeted therapy followed by 2L immunotherapy/none (HR 0.40, 95% CI: 0.24–0.67, *P* < .001), better OS (HR 0.49, 95% CI: 0.30–0.81, *P* = .005), and reduced incident brain metastases (HR 0.47, 95% CI: 0.20–0.99, *P* = .047; Figure 5 and Table 5).

**TABLE 4:** First‐line and second‐line treatment sequences

	First line treatment	
Second line treatment	Immunotherapy combo (*N* = 72, 24.2%)	Targeted therapy combo (*N* = 225, 75.8%)
Targeted therapy BRAFi	1 (1.4%)	N/A
Targeted therapy combo	18 (25.0%)	N/A
Immunotherapy anti‐CTLA4	N/A	8 (3.6%)
Immunotherapy anti‐PD1	N/A	51 (22.7%)
Immunotherapy Combo	N/A	33 (14.7%)
None	53 (73.6%)	133 (59.1%)

**TABLE 5:** Matching weighted comparative effectiveness for sequencing strategies across endpoints

Ref: Targeted combo → Immunotherapy single/combo/none	Hazard ratio (95% CI, *P*‐value)
Immunotherapy combo → Targeted single/combo/none	
Brain metastasis free survival	0.40 (0.24–0.67, *P* < .001)
Overall survival	0.49 (0.30–0.81, *P* = .005)
Incident brain metastases	0.47 (0.20‐0.99, *P* = .047)

## 4 DISCUSSION

Therapeutic advances in the past decade have transformed the care and clinical outcome of patients with advanced melanoma. In this retrospective study, we concluded that patients with stage III or IV BRAF mutant melanoma treated with 1L immunotherapy had significantly longer BMFS and OS compared with those treated with 1L targeted therapy. These conclusions agree with other retrospective analyses, which analyzed fewer patients.^35^, ^36^ Median OS of combination immunotherapy in our analysis of real‐world data was 48.3 months (95% CI: 27.4–NR; Figure S1). Extended follow up data for the Checkmate 067 trial recently reported that the median OS for combination immunotherapy (ie, nivolumab and ipilimumab) was greater than 60.0 months (median not reached; 95% CI, 38.2–NR).^37^ Unadjusted comparisons of the 1 and 2 years OS in the Checkmate 067 (based on graphics capture^38^ and inversion of Kaplan–Meier equations^39^ and Flatiron cohorts) did not show evidence of OS differences (*P* = .478 and *P* = .952, respectively). Median OS of combination targeted therapy in our analysis of real‐world data was 16.0 months (95% CI: 12.6–19.4; Figure S1). While our results are similar to other data reported outside of a clinical trial setting,^35^, ^40^ extended follow up data for the Combi‐d trial reported that the median OS for the combination of dabrafenib plus trametinib was significantly greater at 25.1 months (95% CI was only reported for the HR).^41^ These observed OS differences between the trial results and our retrospective analyses may be due to trial inclusion requirements, such as good performance status and limited comorbidities, which are not present in real‐world patients.^42^ However, the differences may also be due to use of targeted therapy for patients with higher disease burden.

Patients who develop brain metastases are exceptionally difficult to treat and have a relatively poor outcome. We observed that melanoma patients receiving 1L targeted therapy were more likely to develop brain metastases whereas patients receiving 1L single agent or combination immunotherapy had prolonged BMFS compared with patients receiving targeted therapy. Patients receiving 1L immunotherapy also had reduced incidence of brain metastases compared with patients receiving 1L targeted therapy (Figure S5). Our results are in agreement with a recent retrospective study of 293 patients, which demonstrated that immune checkpoint blockade more effectively prevents the development of brain metastases compared with other therapies.^43^

We further analyzed a subset of patients that received 1L combination immunotherapy followed by 2L targeted therapy (including combination targeted therapy and single agent BRAF inhibition) or no 2L therapy as well as those patients who received 1L combination targeted therapy followed by 2L immunotherapy (including combination immunotherapy and single agent CTLA4 or PD1 immunotherapy) or no 2L therapy. Patients receiving 1L combination immunotherapy followed by 2L combination targeted therapy had improved BMFS and OS relative to patients receiving 1L combination targeted therapy even when followed by 2L combination immunotherapy.

While our results are highly significant and strongly support the use of immunotherapy in the 1L setting, the data were evaluated retrospectively and there are several limitations to this type of analysis. Information related to disease burden, extent of metastasis, screening criteria for detection of brain metastases, and other therapies (eg, surgery, radiation, etc.), is unknown. We attempted to control for treatment bias by comparing treatments conducted within specific time windows that reflect widespread availability and adoption of both agents. There were differences in the distributions of follow‐up times between the immunotherapy and targeted therapy cohorts, which were likely driven by the differences in survival times between the immunotherapy and targeted therapy groups, as well as treatment uptake imbalances over time (see Table 1). In the present analyses, only patients with a potential for 6 months of follow‐up were included, and each patient's date of 1L initiation was included as a balancing factor in the propensity model. Further, the (inverse propensity weighted) Cox PH and competing risks models only compare patients who are at‐risk at the same follow‐up duration from 1L initiation. Propensity distributions were strongly overlapping, indicating comparability of immunotherapy and targeted therapy. Subgroup comparisons suggested that the benefit of immunotherapy may be greater for patients with a greater propensity for receiving immunotherapy, suggesting that providers may be using patient characteristics to prescribe immunotherapy to those who are most likely to receive the greatest benefit. Prior to clinical implementation, further validation of these findings is needed. Based on our retrospective analysis, we anticipate that 1L immunotherapy will be superior to 1L targeted therapy not only in regards to BMFS but OS as well. With the recent approval of adjuvant therapy in stage II and III patients, future studies that evaluate the ability of these therapies to improve OS and prevent brain metastases are warranted.

## CONFLICT OF INTEREST

The authors declare no conflict of interest.

## AUTHOR CONTRIBUTIONS

All authors had full access to the data in the study and take responsibility for the integrity of the data and the accuracy of the data analysis. *Conceptualization*, X.W., B.H., S.H., H.C., and S.L.H.; *Methodology*, X.W., B.H., S.H., H.C., and S.L.H.; *Formal Analysis*, X.W., B.H., S.H., H.C., and S.L.H.; *Writing—Original Draft*, X.W.; *Writing—Review & Editing*, B.H., S.H., H.C., and S.L.H.; *Supervision*, B.H., S.H., H.C., and S.L.H.; *Data Curation*, X.W.

## ETHICAL STATEMENT

Institutional review board (IRB) approval of the study protocol was obtained prior to study conduct. The IRB exempted the researchers from obtaining patient consent based on the retrospective and non‐interventional nature of the study (protocol number 00110836).

## Supporting information


**Appendix** S1: Supporting information

## Data Availability

The data utilized in this study were originated by Flatiron Health, Inc. These de‐identified data are subject to a license agreement with Flatiron Health and may be made available upon request; to determine licensing terms, contact DataAccess@flatiron.com.
