# Supplementary material for: First line immunotherapy extends brain metastasis free survival, improves overall survival, and reduces the incidence of brain metastasis in patients with advanced melanoma
Source: Cancer Rep (Hoboken). 2021 Jun 17;4(6):e1419. doi: 10.1002/cnr2.1419 (PMC8714542; doi:10.1002/cnr2.1419)
Supplement: Supplementary file 1 — Appendix S1: Supporting information [file CNR2-4-e1419-s001.docx]

**Appendix**

| 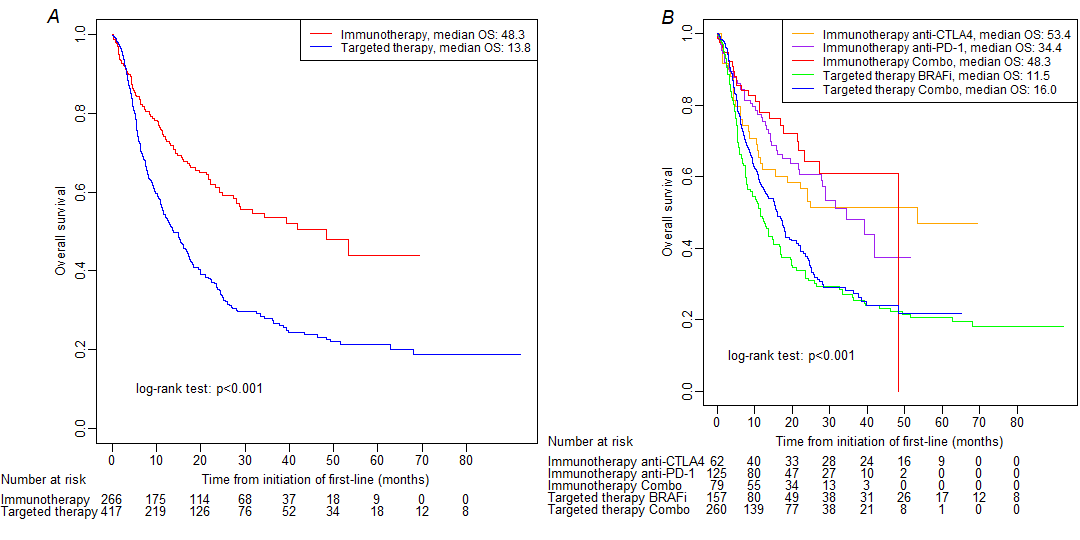  Figure 1. Kaplan-Meier curves of overall survival for BRAF mutated patients who didn’t have brain metastases at 1L initiation by first-line treatments (A), and by break-down of first-line treatments (B) |
| --- |

| 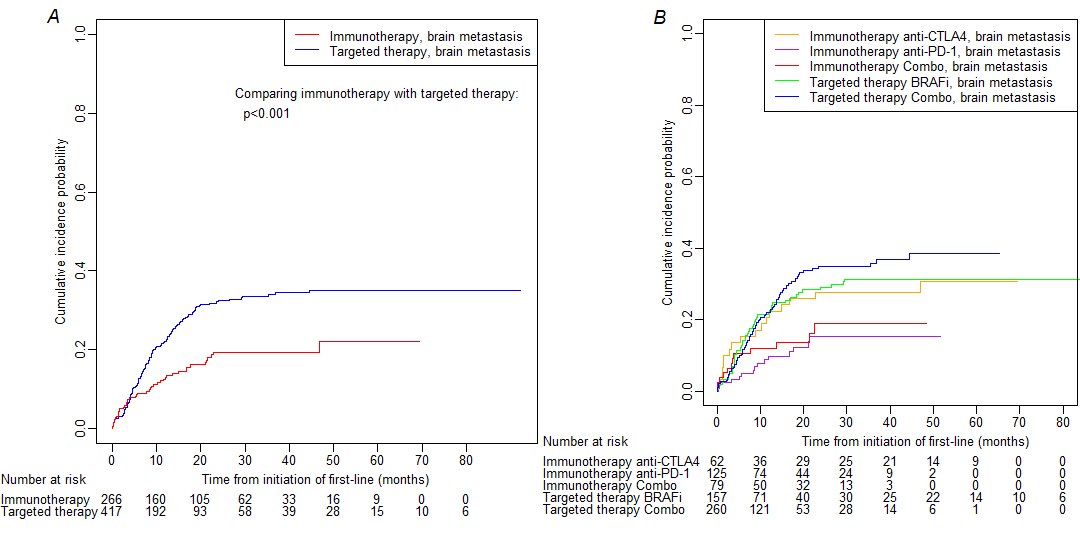  Figure 2. Cumulative incidence probability of brain metastasis free survival for BRAF mutated patients who didn’t have brain metastases at 1L initiation by first-line treatments (A), and by break-down of first-line treatments (B) |
| --- |

| 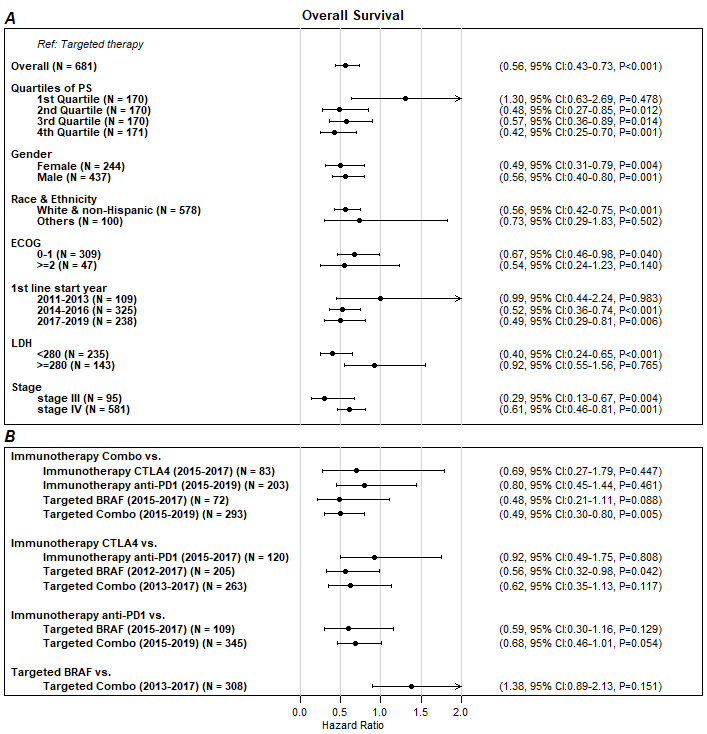  (Note: PS is the probability of receiving immunotherapy.)  (The date ranges of the figure correspond to when both treatments of the comparisons are available.)  Figure 3. Forest plots of hazard ratios of overall survival from matching weighted Cox PH models for subgroups of patients who did not have brain metastases at 1L initiation (A), and for pairwise of treatment regimens (B) |
| --- |

| 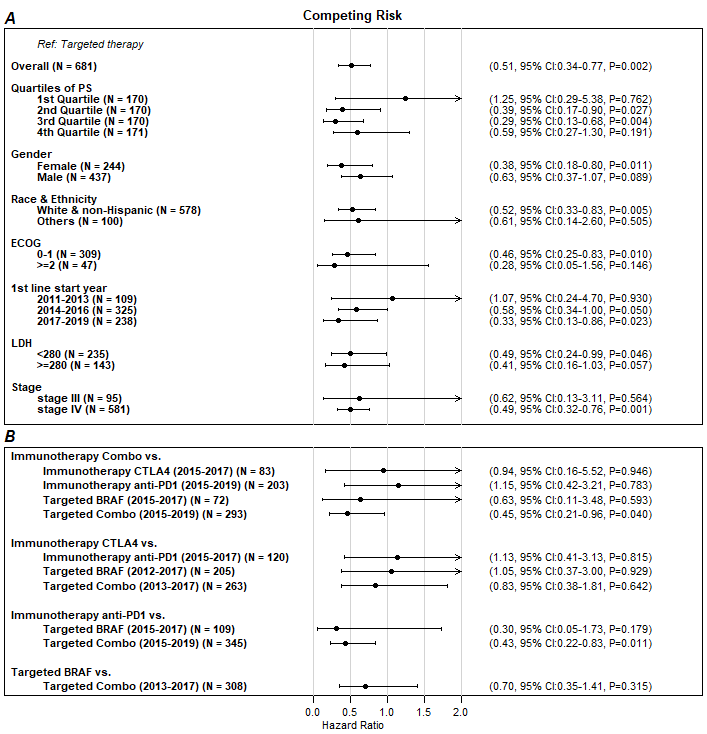  (Note: PS is the probability of receiving immunotherapy.)  (The date ranges of the figure correspond to when both treatments of the comparisons are available.)  Figure 4. Forest plots of hazard ratios of brain metastasis from matching weighted competing risk regression for subgroups of patients who didn’t have brain metastases at 1L initiation (A), and for pairwise of treatment regimens (B) |
| --- |

| 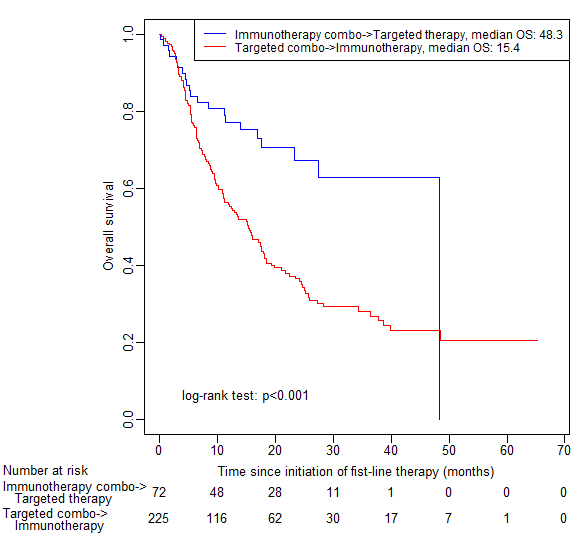  Figure 5. Kaplan-Meier curves by treatment sequences for overall survival |
| --- |

| 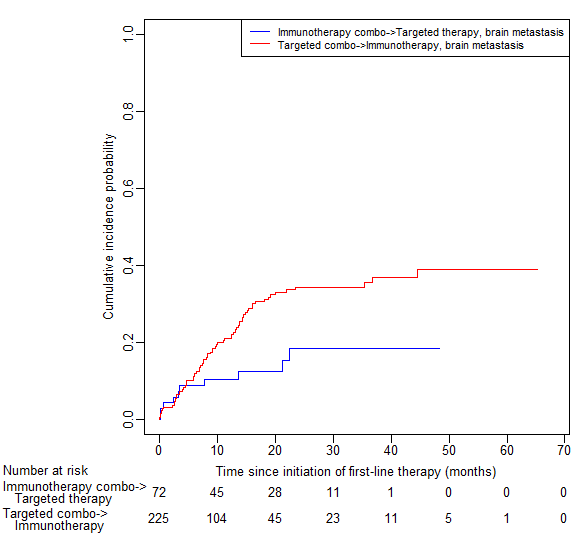  Figure 6. Cumulative incidence probability of brain metastasis free survival by treatment sequences |
| --- |
